# Emergency Severity Index: accuracy in risk classification

**DOI:** 10.1590/S1679-45082017AO3964

**Published:** 2017

**Authors:** Joselito Adriano da Silva, Angélica Santos Emi, Eliseth Ribeiro Leão, Maria Carolina Barbosa Teixeira Lopes, Meiry Fernanda Pinto Okuno, Ruth Ester Assayag Batista

**Affiliations:** 1 Hospital Israelita Albert Einstein, São Paulo, SP, Brazil.; 2 Universidade Federal de São Paulo, São Paulo, SP, Brazil.

**Keywords:** Triage, Emergency medical services, Risk assessment, Emergency nursing, Protocols, Triagem, Serviços médicos de emergência, Medição de risco, Enfermagem em emergência, Protocolos

## Abstract

**Objective:**

To verify agreement between estimative of predicted resources using the adapted Emergency Severity Index and the real amount of resources used by patients. To analyze the variables number of years since graduation, years of work experience and years of experience in emergency services especially with accurate anticipation of resources need.

**Methods:**

This retrospective analytical study with a quantitative approach included 538 medical records of patients assisted by 11 triage nurses. Data collected were related to assistances carried out from December 2012 to February 2013.

**Results:**

There was no significant association between the adequacy of the number of resources used, based on Emergency Severity Index score, number of years since graduation, year of work experience or years of experience in emergency services. Kappa agreement coefficient (0.34) showed that agreement was low between predicted and real used number of resources.

**Conclusion:**

Nurses’ accuracy index to predict resources for patients care from emergency room using the adapted Emergency Severity Index was lower than results reported in the studies in the literature that used the original scale. There was low agreement of diagnostic exams predicted by nurses and those really performed. There was no association among correct prediction of resources needed, number of years since graduation, years of experience in emergency services and years of work experience in the unit where the study was done.

## RESUMO

**Objetivo:**

Verificar a concordância entre a estimativa de recursos previstos por meio do Índice de Gravidade de Emergência adaptada e aqueles utilizados pelos pacientes; e analisar as variáveis tempo de formado, experiência profissional e tempo de trabalho nos serviços de emergência com a assertividade da previsao de recursos.

**Métodos:**

Estudo retrospectivo analítico com abordagem quantitativa. A amostra foi de 538 prontuários de pacientes atendidos por 11 enfermeiros responsáveis pela classificação de risco. A coleta de dados foi realizada em relaçao ao período de atendimento de dezembro de 2012 a fevereiro de 2013.

**Resultados:**

Não houve associação significativa da adequação do número de recursos utilizados, de acordo com a classificação do Índice de Gravidade de Emergência, com o tempo de formação, de experiência ou de trabalho nos serviços de emergência. O coeficiente de concordância Kappa (0,34) demonstrou que a concordância entre o número previsto e o utilizado foi baixa.

**Conclusão:**

Os índices de assertividade dos enfermeiros na previsão de recursos, no atendimento de pacientes na unidade de pronto atendimento, utilizando o Índice de Gravidade de Emergência adaptado, foram inferiores aos descritos na literatura com a escala original. Houve baixa concordância dos exames diagnósticos previstos pelos enfermeiros com os realizados. Não foi observada associação na previsão correta do número de recursos com o tempo de formação, tempo de experiência nos serviços de emergência e tempo de trabalho na unidade de estudo.

## INTRODUCTION

The overcrowding in the emergency services is a global public health problem that has worsened in the last decades. This is a multifactorial problem and possible reasons are deconstruction of primary care network, increase in health services demand, decrease in number of hospital beds and insufficient physicians and nurses to manage all deliveries.^(^ ^1^ ^)^ These facts lead to disappointment of patients because of the long waiting time, and they can compromise the delivery of rapid care to more complex cases, and also can contribute to increase patients’ mortality.^(^ ^2^ ^)^

In 2004 the Brazilian Ministry of Health's Program for Humanization implemented the User Embracement with Evaluation and Risk Classification (AACR - *Acolhimento com Avaliação e Classificação de Risco*) in emergency services. This action was created to promote quality in health care, commitment, dignity and respect to all individuals who seek emergency services. In addition, this action states that care should be organized based on patients’ level of severity rather than order of arrival. As a consequence, to prioritize a patient who needs urgent care increases the satisfaction of other patients, reduces crowding, organizes care flow and promotes a better use of resources.^(^ ^3^ ^)^

The AACR goal is to improve access to health services and promote changes. These changes aim at enhancing relationships between health professionals and patients regarding how care is delivered to them by the use of an attentive listening and consideration of patients based on their risk level, higher integrity among health team members, and provision of care with higher responsibility and safety.^(^ ^4^ ^)^ To implement AACR in emergency units can reduce risks, prevent deaths, exclude triage done by non-qualified health professionals, prioritize patients care based on clinical criteria, reduce waiting time, identify cases that can compromise late care, promote adequate care by reducing risks and increasing safety, and manage resources to be used by patients.^(^ ^5^ ^)^

The AACR should be performed by graduated health professionals who had received specific training and by the use of pre-establish protocols.^(^ ^3^ ^)^

According to guidelines of national policies for humanization, the AACR must be done by a nurse who are trained to perform this role.^(^ ^3^ ^)^ The professional regulations also support to perform such activity because nursing consultation and prescription are private activities of nurses, and risk classification is part of nursing consultation.^(^ ^5^ ^)^

Generally, the use of protocols has been recommended to stratify risk in five levels because they present more reliability and validity to assess patients’ clinical status. Currently, the commonly used protocols around the world to classify risk in urgent/emergency services are: Australasian Triage Scale (ATS), Canadian Triage & Acuity Scale (CTAS), Emergency Severity Index (ESI) and Manchester Triage System (MTS). All these protocols uses standardize triage acuity scales including five levels of priority.^(^ ^6^ ^)^

No standard scale exists for health care measurement. Each institution has autonomy to use the most adequate scale for its needs. Health care measurement requires indicators that represent a concept. No measurement instrument is free of fails, situational and environmental factors, biased responses, personal influence and changes in data collection that can lead to errors in measurement.^(^ ^7^ ^)^

In ESI scale, patients are classified and prioritize based on level of severity of their disease by estimating the number of resources need for his/her care. Less severe patients often require fewer resources and they are classified as ESI level 5 whereas more severe patients need four or more resources, and they are classified as level 1. Patients classified as level 1 need immediate medical care, patients classified as level 2 and 3 need care within 15 minutes, and those classified as level 4 and 5 need care within 30 minutes.^(^ ^8^ ^)^

The ESI objective is to decentralize medical care. In a first analysis, focus would be to provide adequate medical care for low-complexity patients who corresponded to levels of 3, 4, and 5. These patients often use fewer resources and, rarely, need hospitalization. Priority is defined based on a flowchart with assessment of resources needed for adequate care, and by assuring that patients have access to resources in the right time.^(^ ^8^ ^)^

Improvement of risk classification is the objective of emergency services because it improves clinical results, mainly in services with limited resources. An international study including different countries showed that ESI is one of the best methods to define care priority based on time of arrival, as well as to be used after treatment to improve access for medical care and available resources.^(^ ^9^ ^)^

## OBJECTIVE

To verify agreement between estimated resources and those really needed based on the an adapted version of the Emergency Severity Index, and analyze variables number of years since graduation, years of work experience and years of experience in emergency with adequate estimation of resources.

## METHODS

This retrospective analytical study with quantitative approached was carried out in Ibirapuera outpatient unit of the *Hospital Israelita Albert Einstein.* This unit woks 24 hours a day and deliveries, on average, 7,150 care each month in different specialties such as clinical medicine, general surgery and pediatrician. In addition, the unit also provides orthopedic consultations.^(^ ^8^ ^)^

The ESI scale was used by nurses to classify risk of patients.^(^ ^7^ ^)^ This scale entails decisive points in which the adequately trained nurse consider the following four questions to classify patients: does the patient need a rapid intervention? Is this a patient who should not wait? How many resources does this patient need? What are his/her vital signs? Patients at risk of death are classified as emergent (level 1 and 2). Other classifications (from level 3 to 5) are based on the number of resources needed (complementary diagnosis test and therapeutic procedures), on vital signs, e.g., complementary diagnostic tests, therapeutic procedures to be used, and vital signs. These levels are: urgency (fast care, level 3), (non urgent, level 4) and non urgent (level 5). The latter two levels are called the supertrack consisting of a system of rooms or fast areas, which aim at the fast protocol resolution of non-severe patients.^(^ ^6^ ^)^

After exclude patients’ risk of death (level 1 and 2) the nurse estimate the number of resources needed for his/her care, such as laboratory tests to support the diagnosis. If patient uses two or more resources, the nurse assess his/her vital sings and, depending on the result, patient can be reclassified as priority level 2.^(^ ^6^ ^)^

The ESI scale was translated into Brazilian Portuguese by nurses and physicians responsible for patients’ care. However, the scale was not validated before implementation. Adequacies were needed during translation process to implement it in the emergency services in the institution where this study was carried out (adapted ESI scale). We did not consider as resources administration of intramuscular, intravenous and subcutaneous medications. Dressing materials and inhalation solution were considered resources only a second application was needed. Procedures not performed in the emergency services were excluded from our analysis, such as complex procedures, mild sedation, and heparinization or catheter salinization (Chart 1).

**Chart 1:** Classification of predicted resources by adapted version of Emergency Severity Index

Resources	ESI	Adapted ESI
Laboratorial tests	Yes	Yes
Rapid tests	No	No
Electrocardiogram, ultrasonography, and radiologic tests	Yes	Yes
Intravenous hydration	Yes	Yes
Intravenous or intramuscular drugs	Yes	No
Consultation with specialists	Yes	Yes
Simple procedures (sutures and urinary catheterization delay)	Yes	Yes
Complex procedures (mild sedation)	Yes	N/A
Intravenous drugs (time of infusion)	Yes	Yes
Subcutaneous and intravenous drugs without time of infusion	Yes	No
Dressings	No	Yes
Inhalation (only in case of second dose)	N/A	Yes
Anamnesis and physical exam	No	No
Heparinization and catheter salinization	No	N/A
Oral drug, immunization against tetanus and changes in medical prescriptions	No	No
Call for principal physician	No	No
Crutch, casts and simple immobilization	No	No

N/A: not applicable.

In this study, patients who were classified as fast track (fast care) were followed by specific area in emergency services and counted on an exclusive team to perform activities. For this reason, a restructuration of infrastructure was done to support the needs of the triage flows.

Electronic data were included risk classification of patients, assisted by an internist, from both sexes who were aged equal or older than 18 years, and classified in level 1-5 of adapted ESI scale.

Sample calculations were done considering a precisely estimation of 5% of an assertive proportion of 70% with significance level of 5%. The sample was included 538 medical records from patients who received care from 11 nurses based on risk classification.

Data collection used information from patients who received care from December 2012 to June 2013. We used a variable of interest form including initial complaint, resources proposal by the adapted ESI, resources used and identification of responsible for the triage. Information were collected from electronic medical record of the hospital management system after obtaining consent from nurses who from site where this study was performed.

Categorical variables were described by summary measures using absolute and relative frequencies (percentages), quantitative variables as means, standard deviations (SD) or median, first and third quartiles (1ºQ; 3ºQ), and also minimal and maximal values.

Number of predicted resources and number of resources used were typed into a double-entry table including absolute frequencies and relative percentages of the total sample. We used Cohen's kappa coefficient to assess agreement between methods of measurements.

Evaluation of associated factors and number of resources adequacy based on ESI was carried out using equations models of generalized estimation, and by considering correlation between observations of single nurse at different care times delivery to different patients. Models were adjusted within multinomial distribution using a simple approach.

For statistical analyses we used the Statistical Package of the Social Sciences (SPSS) software, version 17. A significance level of 5% was adopted.

The Institutional Ethical and Research Institute approved this projected, number 650,584, CAAE: 27423314.0.0000.0071.

## RESULTS

Triage was performed by 11 nurses, and most of the them were female (81.8%). Nurses’ age ranged from 29 to 36 years, and number of care delivery done by each of them ranged from 27 to 53 patients’. Most of nurses had less than 7 years since graduation (54.5%) (Table 1).

**Table 1:** Description of nurses’ characteristics

Variable		n (%)
Nurse's age (years)		
	Mean (SD)	32.4(3.8)
	Minimal-maximal	26-38
Sex of nurses, n (%)		
	Female	9(81.8)
	Male	2 (18.2)
Years since graduation		
	Up to 7 years	6 (54.5)
	More than 7 years	5(45.5)
	Median (1°Q; 3°Q)	7 (4;9)
	Minimal-maximal	4-11
Years of experience in emergency services		
	Up to 6 years	6 (54.5)
	More than 6 years	5(45.5)
	Median (1°Q; 3°Q)	6 (4;8)
	Minimal-maximal	4-10
Years of work experience		
	Up to 2 years	6 (54.5)
	More than 2 years	5(45.5)
	Median (1°Q; 3°Q)	2 (2;4)
	Minimal-maximal	1-6
Number of care delivered		
	Median (1°Q; 3°Q)	50 (27;53)
	Minimal-maximal	22-88

SD: Standard deviation; Q: quartile.

Our sample included 538 medical records. Patients’ age ranged from 18 to 65 years, mean of 39.4 years (SD=10.7 years). Of these, 59.3% were female.

Risk classification ranged between 2 and 5 with higher percentage of patients classified in level 3 (61%); 47.4% of patients reported two or more symptoms during care. Participants’ main complaints were nausea and vomiting (22.30%) followed by sore throat (15.24%), abdominal pain (14.30%) and cough (13.01%). Regarding sex, men sought the service more than female and most of patients had only one complaint (Table 2).

**Table 2:** Clinical and demographic variables, emergency severity index according to patients’ medical records

Characteristics		n (%)
Patients’ mean age (SD)		39.4 (10.7)
Female patients'		319 (59.3)
Number of symptoms		
	1	283 (52.6)
	2	166 (30.9)
	3	77 (14.3)
	4	11 (2.0)
	5	1 (0.2)
Level of screening classification		
	2	60 (11.2)
	3	328 (61.0)
	4	145 (27.0)
	5	5 (0.9)
Correspondence between screening diagnosis and discharge		514 (95.5)
Resources used		
	0	116 (21.6)
	1	126 (23.4)
	2 or more	296 (55.0)
Resources predicted by ESI		
	0	5 (0.9)
	1	145 (27.0)
	2 or more	388 (72.1)
Adequacy of number of resources based on ESI		
	Lower than recommended level	181 (33.6)
	Adequate	348 (64.7)
	Higher than recommended level	9 (1.7)

SD: standard deviation; ESI: Emergency Severity Index.

Level 3, the corrected prediction of resources was 36% and, in level 4, cases not requiring resources or specific procedures the resulted was 37%.

Accuracy between number of resources predicted and used was 64.7% (Table 3). The Kappa coefficient agreement was 0.34, *i.e.,* the agreement between number of predicted resources and number of used resources was low and most common discordances were cases that used fewer resources than the total predicted in the assessment using the ESI scale.

**Table 3:** Resources used and predicted using the Emergency Severity Index scale

Resources used and predicted		n (%)
Adequate prediction		
	None predicted/none used	5 (0.9)
	Predicted/used	56 (10.4)
	Two or more predictions/two or more used	287 (53.3)
Number of resources lower than predicted		
	One predicted/none used	80 (14.9)
	Two or more predicted/none used	31 (5.8)
	Two or more predicted/one used	70 (13.0)
Number of resources higher than prediction		
	None predicted/only one used	0 (0.0)
	None predicted/two or more used	0 (0.0)
	One predicted/two or more used	9(1.7)
Total		538 (100)

No significantly associated evidences were observed in terms adequacy of number of resources used according to ESI scale based on explicative variables (Table 4).

**Table 4:** Association between adequacy of number of resources used according to Emergency Severity Index scale and variables of interest

Characteristics of nurses		Adequate number of resources based on p value according to ESI classification			p value
		Lower than recommended	Adequate	Higher than recommended	
Years since graduation, n (%)					
	Up to 7 years (n=345)	122(35.4)	220 (63.8)	3 (0.9)	0.100
	More than 7 years (n=193)	59 (30.6)	128 (66.3)	6(3.1)	
	Up to 6 years (n=352)	120 (34.1)	229 (65.1)	3 (0.9)	0.698
	More than 6 years (n=186)	61 (32.8)	119 (64.0)	6 (3.2)	
Years of work experience in emergency services, n (%)					
	Up to 2 years (n=250)	80 (32.0)	168 (67.2)	2 (0.8)	0.471
	More than 2 years (n=288)	101 (35.1)	180 (62.5)	7 (2.4)	

ESI: Emergency Severity Index.

## DISCUSSION

The Brazilian National Humanization Policy recommends to implement in emergency services an user embracement approach along with risk classification.^(^ ^3^ ^)^ Agreement between professional's assessment and institutional protocols is critical to guarantee safety of care delivery.^(^ ^7^ ^)^ A study developed in Iran compared two triage system and observed that ESI had important function to prioritize care because it classify patient in the right order, right time and predict the correct number of resources need.^(^ ^10^ ^)^

A study in a public hospital in the city of São Paulo evaluated nurses who did risk classification and reported similar results to our study. Most of participants were female (90.0%) with mean age of 27.3 years. However, years since graduation in our study (5 years) was greater.^(^ ^11^ ^)^ The study also reported that years of experience in urgent and emergency services contributed to accuracy of risk classification.^(^ ^12^ ^)^

Characteristics of population identified in the emergency services was little complexity patients who could be easily assisted in the health basic care unit (outpatient units). This finding is similar to those reported in the literature.^(^ ^13^ ^)^ Our results show the inadequate use of emergency services by population and this could be the reason of units overcrowding by individuals who could be assisted at low-complexity services. Main reasons cited in literature for patients sought for emergency services are efficient care, availability of medicines and sophisticated laboratorial tests.^(^ ^14^ ^)^

Complaints that were higher frequent among patients were nausea and vomiting, followed by sore throat, abdominal pain and cough. A study carried out in Minas Gerais using Manchester scale showed that most used flowcharts were “malaise in adults” that is used when patient does not have a specific complaint and of “palpitation, chest pain and headache”.^(^ ^7^ ^)^

Nurses’ accuracy to predict resources was 64.7%, which is a similar result reported by a study done in teaching and community hospital in the USA that implemented and validated ESI scale and nurses of the study were able to predict resources needed in 70% of cases.^(^ ^15^ ^)^ However, the study also pointed out that agreement was low between number of predicted and used recourses, and most common discordances were patients that used fewer resources than predicted. This results might be due to low death risk among patients because they were individuals presenting low clinical complexity. There is need of follow-up this situation more closely and implement educational interventions to correct it.

A better degree of accuracy was observed in our study in patients classified as level 2. Nurses evaluation had an accuracy of 64.7% in terms of resources adequacy. Similar results were observed in a study that compared agreement in classification between nurses and physicians using ESI scale that reported high percentage of accuracy for levels 1 and 2 (100 and 95%, respectively).^(^ ^16^ ^)^ A study carried out in Switzerland including four emergency services analyzed and classified scenarios according to ESI scale, the authors reported that in ESI level 2 almost half nurses (50.2%) did the rate incorrectly.^(^ ^17^ ^)^ We understand that low accuracy percentage among critically ill patients and any delay in care can increase the risk of death. In other study, carried out in Belgium, the nurses’ accuracy according to ESI scale in a simulated situations was 77.58%, however variations were observed when levels of severity were evaluated separately. In addition, regarding resources adequacy classification below than recommended was the most common, and ESI level 2 had higher percentage of errors, and 99.20% of simulated situations were classified below the recommended.^(^ ^18^ ^)^

Based on ESI scale, patients with pain score higher than 7 could be classified in level 2. However, nurses are recommend to correlate this score with other parameters, such as changes in vital sings and patient's behavior in triage. However, the pain assessment stills a challenge, and professional should consider clinical features, stage of the disease, and the ability of patient to communicate. Therefore, when a nurse opts to measure pain using scales, he/she must consider other vital parameters as well.^(^ ^19^ ^)^

In level 3, nurses’ accuracy was 36% most of them were not able to predict number of resources used for each patient. In level 4, we also observed that a variety of cases did not require resources or specific procedures, however, some resources were used (37%). Regarding these two levels the study from Switzerland reported different results and an accuracy rate of 70%.^(^ ^17^ ^)^

Characteristics of nurses were not significantly associated with adequacy of number of resources. Perhaps this result was due to existence of a preestablished protocol to guide and uniform actions during risk classification.

We highlight the importance of further studies in the subject associated with care quality and safety. Limitations of our studies include data collection carried out in a single center, the use of a retrospective approach, which might restricted findings to a local reality. To date, few studies assessed validity and reliability of protocols to determine risk of patients in emergency services. Emergency Severity Index is an American scale and no studies in Brazil have been done to translate and validate it. For this reason, the scale reliability can be weak because its analytical performance was not previously tested among Brazilian population. Further studies are warranted to verify accuracy of using this scale to predict resources used in national screening services.

In our study nurses’ accuracy was 64.4% using an adapted scale ESI. This finding can indicate the need of regular refresher trainings in ESI application, and reassessments of the process to increase patient safety and bring benefits to health care institutions.

## CONCLUSION

In our study, nurses’ accuracy to predict resources for patients care in emergency service using the adapted ESI were lower than results reported in the literature that used the scale in its original version. A low agreement was observed between number of estimated and used resources.

No association was seen regarding correct prediction of resources and years since graduation, years of work experience in emergency unit, and also years of work experience in the institution where the study was done. To achieve excellence in health care services is a trend to improve patient safety. For this reason, it is important to highlight the need of analysis of triage process and provision of regular refresher training for health professionals involved in such process.
